# Food Protein-Induced Enterocolitis Syndrome Causing Hypovolemic Shock and Methemoglobinemia

**DOI:** 10.1155/2018/1903787

**Published:** 2018-04-30

**Authors:** Stefan W. Malin, Riad Lutfi, Matthew L. Friedman, Alicia M. Teagarden

**Affiliations:** ^1^Department of Pediatrics, Indiana University School of Medicine, Indianapolis, IN, USA; ^2^Division of Pediatric Critical Care, Department of Pediatrics, Indiana University School of Medicine, Indianapolis, IN, USA

## Abstract

A 5-week-old previously healthy male presented with vomiting and diarrhea leading to hypovolemic shock and profound metabolic acidosis. He was subsequently found to have severe methemoglobinemia. The acidosis and shock improved with fluid resuscitation and methemoglobinemia was successfully treated with methylene blue. An extensive workup, including evaluations for infectious and metabolic etiologies, was unremarkable. However, a detailed dietary history revealed a recent change in diet, supporting a diagnosis of food protein-induced enterocolitis syndrome (FPIES). We present this case to highlight the importance of considering FPIES in an infant with vomiting and diarrhea, in the setting of a recent dietary change, leading to profound dehydration, metabolic acidosis, and methemoglobinemia. Diagnosis of FPIES, although difficult to make and one of exclusion, can be potentially life-saving.

## 1. Introduction

Non-IgE-mediated food allergies can have acute or chronic presentations [1]. Food protein-induced enterocolitis syndrome (FPIES) is a rare, though severe, form of non-IgE-mediated food allergy that can present in infancy with predominant gastrointestinal signs and profound dehydration. Severe methemoglobinemia in the setting of acute intestinal inflammation and increased intestinal nitrites has been described infrequently in FPIES [2]. Methemoglobinemia is a blood disorder in which the ratio of oxidized hemoglobin is altered resulting in a shift in the oxygen hemoglobin dissociation curve. Rapid recognition of FPIES and appropriate therapy is crucial to avoiding recurrent hospitalizations, to unnecessary extensive workup, or in extreme cases, even death.

## 2. Case Presentation

A five-week-old previously healthy, full-term male initially presented to an emergency department with a two-day history of profound vomiting and diarrhea. He was well appearing and afebrile at the time of presentation; reassurance was given and he was discharged home with close follow-up. At his follow-up appointment, an upper gastrointestinal series (UGI) was obtained that was reassuring. His symptoms persisted, however, so, roughly 24 hours following his UGI, he was taken to another emergency department for worsening gastrointestinal symptoms and lethargy. His initial blood gas showed severe metabolic acidosis with a pH of 6.7, bicarbonate of 3 mmol/L, and base deficit exceeding −30 mmol/L. Intraosseous access was obtained prior to transport to the tertiary care pediatric facility and the child received 20 ml/kg of normal saline during transport.

**Figure 1:**
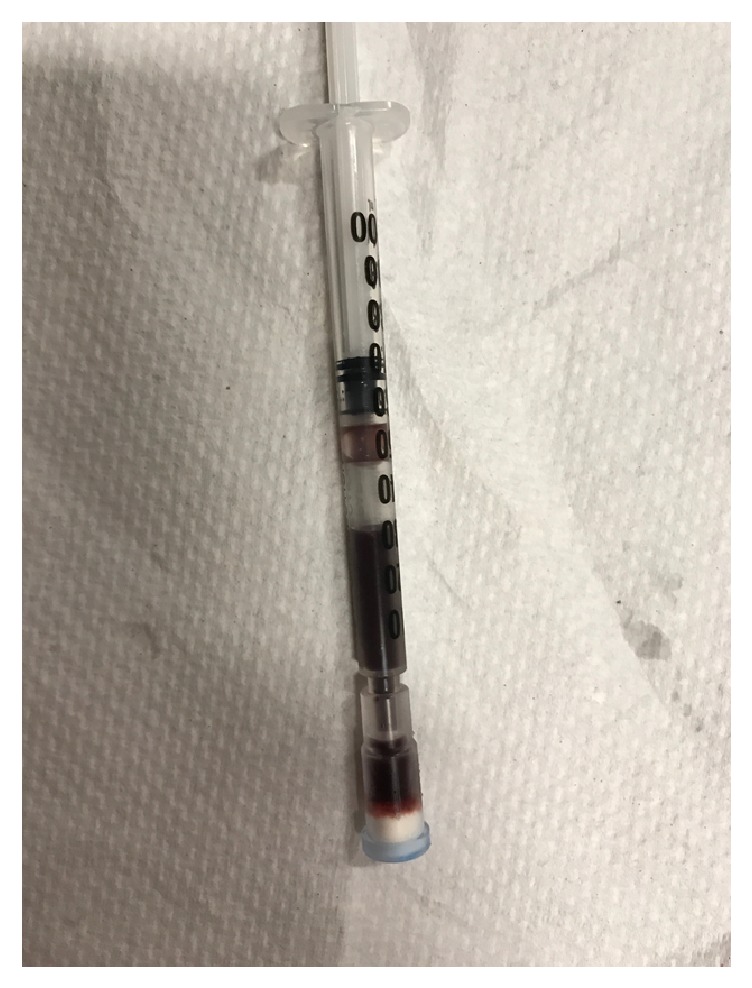
This is an arterial blood sample obtained shortly after admission. It was noted to be significantly darker than expected. Cooximetry was obtained and the methemoglobinemia level was reported as greater than 25%.

Upon arrival to the pediatric intensive care unit, the child was lethargic, cyanotic, and mottled with cool extremities. His exam was notable for decreased muscle tone, labored breathing, a soft but distended abdomen, prolonged capillary refill, and a sunken anterior fontanelle. His initial heart rate was 150 beats per minute, blood pressure was 70/30 mmHg, and oxygen saturation was 85% on 2 liters/minute nasal cannula. Intravenous access was obtained and the patient received aggressive isotonic crystalloid resuscitation. He was intubated due to severe acidosis and altered mental status. A broad workup was initiated after stabilization including evaluation for infectious, metabolic, and cardiac causes. His initial labs were also notable for an elevated white blood cell count of 51 k/cumm (normal 6–18), with 38% bands, 5% myelocytes, and 8% metamyelocytes, an ammonia of 211 mCmol/L (normal 11–35), and a lactate of 1.8 mmol/L (normal 0.5–1.6). Stool hemoccult was positive. He was placed on broad-spectrum antibiotics pending blood, urine, cerebrospinal fluid, and stool cultures. An echocardiogram was obtained that demonstrated normal anatomy and function. The child was persistently hypoxemic after intubation despite receiving 100% fraction of inspired oxygen on the ventilator, so an arterial blood gas was obtained and the blood was noted to be dark chocolate colored in appearance (Figure 1). The partial pressure of oxygen was 200 mmHg. Cooximetry was ordered with a serum methemoglobin level, which was greater than 25%, consistent with the diagnosis of methemoglobinemia (>1.5% is considered elevated per laboratory equipment and protocol). After two doses of 1 mg/kg of methylene blue, his serum methemoglobin level decreased to 3.5%. His ammonia level and acidosis corrected with fluid resuscitation. He was extubated roughly 18 hours after presentation.

**Figure 2:**
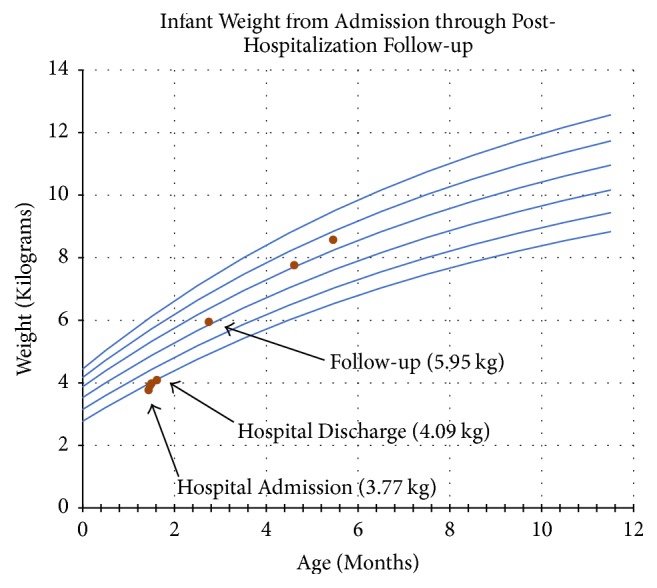
This is a copy of the infant's growth chart. The infant was admitted to the PICU and was 3.77 kilograms (kg), which was below his birth weight and at the 6th percentile for age. He was discharged weighing 4.09 kg and at posthospitalization follow-up (6 weeks from discharge) he was 5.95 kg, approximately the 52nd percentile for age.

Multiple subspecialties were consulted to determine the cause of his severe metabolic acidosis and methemoglobinemia, including Metabolism and Gastroenterology. At this point, further history revealed that the infant's formula had been changed multiple times in the days preceding presentation. Malabsorptive causes were considered unlikely due to normal growth prior to presentation and allergic enteritis/proctitis was considered unlikely due to the severity of presentation. Eosinophilic esophagitis was not considered in the differential from gastroenterology because of the diarrhea and severity of the presentation. Infectious enteritis and colitis were initially presumed to be the most likely cause, but a stool culture, ova and parasite exam, rotavirus antigen, and gastrointestinal pathogen PCR test were all negative. The infant was started on hydrolyzed formula (Elecare) and discharged home with follow-up appointments with a pediatric gastroenterologist and an allergist with a presumed diagnosis of FPIES. At the time of his follow-up, roughly 8 weeks after discharge, he was continuing to do well on Elecare and had not had recurrence of his symptoms, and his weight had returned to the 52nd percentile up from the 6th percentile at his initial presentation (Figure 2). His diet has slowly been expanded with the exception of milk; he will have to complete an oral food challenge (OFC) with milk in a controlled office setting prior to its reintroduction.

## 3. Discussion

Pediatric food allergies are common in the first two years of life and have an estimated prevalence between 3 and 8% [1]. FPIES, a non-IgE-mediated food allergy, had previously been considered a rare disorder, but due to raised awareness, recent studies have estimated its incidence at up to 0.3% of all newborns [1, 3]. Food protein-induced allergic proctocolitis and food protein-induced enteropathy are also non-IgE-mediated food allergies, but they often have subacute to chronic presentations ranging from hemoccult-positive stools in healthy infants to chronic diarrhea causing failure to thrive [1]. Differentiation between the various non-IgE-mediated food allergies is made based on clinical history and severity of illness. Occasionally, endoscopies with biopsies are required for diagnosis [1]. FPIES is a severe form of food hypersensitivity reaction that usually presents in infancy and is often difficult to recognize and diagnose [3, 4]. This will hopefully improve, especially considering that the first international evidence-based guidelines for the diagnosis of FPIES were recently published in 2017 [1]. FPIES can present with varying degrees of severity, but at its worst, can present with profuse vomiting, diarrhea, dehydration, and eventual lethargy and shock [4]. At its least severe form, unrecognized FPIES can lead to failure to thrive and potentially multiple admissions with extensive testing and interventions [1, 3]. Increased awareness of FPIES can hopefully lead to improved recognition in subacute cases and need for referral to a tertiary care center in severe cases.

The precise pathogenesis of FPIES has not been clearly defined. T-cells are often considered as a major role player; however, this has come under recent scrutiny [1, 5]. Studies have shown similar CD4 T-cell responses after casein exposure in cow-milk FPIES patients as compared to IgE-mediated allergy to cow's milk and normal subjects [5]. Activation of the innate immune system has recently been identified as a potential role player in FPIES. Monocytes, natural killer cells, neutrophils, and eosinophils, identified by whole blood flow cytometry, were activated in patients with active FPIES exposed to milk proteins [6]. This same pattern was not observed in children who had outgrown their FPIES [6]. Growth of local populations of plasma cells in the intestine has been seen in biopsies following acute FPIES reactions resulting in increased production of IgM and IgA [7]. Only 10–30% of patients will have a positive IgE against the offending food [7]. FPIES often results in a systemic inflammatory response not seen in other non-IgE-mediated food allergies and can present with a profound leukocytosis, margination of leukocytes, and a markedly elevated C-reactive protein [1]. Cytokine profiles in patients with positive OFCs were notable for elevations in IL-2, IL-5, and IL-8, and other cytokines were markedly elevated in patients depending on the severity of their reaction [8]. Despite the fact that the exact etiology is unknown, the end result is severe intestinal inflammation leading to profuse diarrhea and vomiting.

Decreased catalase activity during acute intestinal inflammation leads to increased intestinal nitrites and can cause increased heme molecule oxidation, which leads to methemoglobinemia [3]. Methemoglobin is unable to bind oxygen and shifts the oxygen-dissociation curve to the left [9]. Infants are more at risk for developing methemoglobinemia as fetal hemoglobin is more easily oxidized and their level of cytochrome-b5 reductase does not reach adult values until roughly 4 months of age [9]. In infants with cyanosis and diarrhea, methemoglobinemia should be considered, especially when cyanosis does not improve with supplemental oxygen and arterial blood appears darker than normal [2, 9]. Cooximetry is the most accurate method to reliably measure methemoglobin. Children with severe methemoglobinemia experience marked respiratory distress and altered mental status that would exacerbate with anemia and acidosis leading to further impairment of oxygen delivery and tissue perfusion [9]. Treatment is based on reducing the oxidized iron within the hemoglobin molecule to its ferrous state using 1 mg/kg of intravenous methylene blue [9].

Given that FPIES can present at a very young age, the differential diagnosis for this clinical presentation is varied and includes sepsis, cardiogenic shock, metabolic disorders, and intestinal etiologies. FPIES is a clinical diagnosis, so obtaining a detailed dietary history as part of the evaluation is crucial. Age of presentation, absence of fever, and recurrent episodes of gastrointestinal symptoms are important keys to the diagnosis [1, 10]. Given its variability in presentation, awareness of FPIES and keeping it on the differential is important, even though it is a diagnosis of exclusion.

Treatment of acute FPIES is primarily supportive with fluid resuscitation and close hemodynamic monitoring. Occasionally, vasopressors or inotropes are needed in addition to fluids to treat hypotension. No strong evidence to support the benefit of steroid treatment is available [3]. New, limited evidence has suggested that IV administration of ondansentron (a 5-hydroxytryptamine_3_ antagonist) as an adjunctive therapy has some benefit in acute presentations [10]. Identifying the causal agent and removing it from the child's diet are crucial and remain the mainstay of treatment. The long-term prognosis for FPIES is good as the majority of children will outgrow their food protein intolerance and catch up with their growth [1, 3]. The age of resolution is variable and depends partly on the offending food substance. For cow milk FPIES, resolution around 1-2 years of age has been reported, although it can take longer than this [10].

In our case report, we discuss the case of an infant presenting with hypovolemic shock, severe metabolic acidosis, and methemoglobinemia due to FPIES. Despite extensive evaluation, no other etiologies were found to explain his symptoms and laboratory findings. Early recognition and aggressive management were key elements for shock reversal. His symptoms and failure to thrive resolved after switching to a hypoallergenic, hydrolyzed formula. FPIES should be suspected in infants who present with diarrhea and methemoglobinemia. As discussed previously, early recognition may prevent excessive diagnostic testing, recurrent hospital admissions, and failure to thrive.
